# Identification and Functional Analysis of E3 Ubiquitin Ligase *g2e3* in Chinese Tongue Sole, *Cynoglossus semilaevis*

**DOI:** 10.3390/ani14172579

**Published:** 2024-09-05

**Authors:** Zhongkai Cui, Jun Luo, Fangzhou Cheng, Wenteng Xu, Jialin Wang, Mengjiao Lin, Yuqi Sun, Songlin Chen

**Affiliations:** 1 College of Fisheries and Life Science, Shanghai Ocean University, Shanghai 201306, China; luojun1998828@163.com (J.L.); chengfangzhou0338@163.com (F.C.); wjl6792@outlook.com (J.W.); linmj000@163.com (M.L.); 2 State Key Laboratory of Mariculture Biobreeding and Sustainable Goods, Yellow Sea Fisheries Research Institute, Chinese Academy of Fishery Sciences, Qingdao 266071, China; cuizk@ysfri.ac.cn (Z.C.); xuwt@ysfri.ac.cn (W.X.); sunyuqi2099926838@163.com (Y.S.); 3 Laboratory for Marine Fisheries Science and Food Production Processes, Qingdao Marine Science and Technology Center, Qingdao 266237, China

**Keywords:** *Cynoglossus semilaevis*, *g2e3*, gametogenesis, promoter, gonad

## Abstract

**Simple Summary:**

This research explores the role of the *Cs-g2e3*, a type of E3 ubiquitin ligase, in the gametogenesis of the Chinese Tongue Sole (*Cynoglossus semilaevis*), a marine fish known for its significant sexual dimorphism due to its unique chromosome system. E3 ubiquitin ligases are enzymes that help tag proteins for degradation or other regulatory functions and are involved in various biological processes, including gametogenesis. Our study focused on understanding how *Cs-g2e3* influences gametogenesis—the process by which gametes or germ cells are produced. We found that *Cs-g2e3* was highly expressed in the gonadal tissues of *C. semilaevis*, with its expression peaking at 8 months of age. By using techniques such as RNA interference, we discovered that the knockdown of *Cs-g2e3* in ovarian and testicular germ cell lines significantly downregulated the expression of spermatogenesis-related and oogenesis-related genes. Additionally, we looked at how different transcription factors, which help turn genes on or off, affect the activity of the *Cs-g2e3* promoter. Our findings highlight the crucial role of *Cs-g2e3* in gametogenesis, suggesting its potential as a novel genetic tool that could significantly advance artificial reproduction technologies in aquaculture.

**Abstract:**

Gametogenesis, the intricate developmental process responsible for the generation of germ cells (gametes), serves as a fundamental prerequisite for the perpetuation of the reproductive cycle across diverse organisms. The *g2e3* enzyme is a putative ubiquitin E3 ligase implicated in the intricate regulatory mechanisms underlying cellular proliferation and division processes. The present study delves into the function of G2/M phase-specific E3 ubiquitin protein ligase (*Cs-g2e3*) in gametogenesis in Chinese Tongue Sole (*Cynoglossus semilaevis*). Sequence analysis shows that the *Cs-g2e3* mRNA spans 6479 bp, encoding a 733 amino acid protein characterized by three conserved structural domains: PHD, RING, and HECT—typical of HECT E3 ubiquitin ligases. The predominant expression of *Cs-g2e3* in the gonad tissues is further verified by qPCR. The expression profile of *Cs-g2e3* in the gonads of the Chinese Tongue Sole is analyzed at different ages, and the results show that its expression peaks at 8 months of age and then begins to decline and stabilize. It is noteworthy that the expression level remains significantly elevated compared to that observed during the juvenile period. In situ hybridization shows that the mRNA of *Cs-g2e3* is mainly localized in the germ cells of the ovary and the testis. RNA interference experiments show that the knockdown of *Cs-g2e3* in ovarian and testicular germ cell lines significantly downregulates the expression of key genes involved in oogenesis (e.g., *sox9* and *cyp19a*) and spermatogenesis (e.g., *tesk1* and *piwil2*), respectively. Furthermore, the analysis of mutations in the transcription factor binding sites reveals that mutations within the Myogenin, YY1, and JunB binding sites significantly impact the transcriptional activity of the *Cs-g2e3* gene, with the mutation in the YY1 binding site exhibiting the most pronounced effect (*p* < 0.001). This study contributes to a deeper understanding of the tissue-specific expression patterns of *Cs-g2e3* across various tissues in *Cynoglossus semilaevis*, as well as the potential regulatory influences of transcription factors on its promoter activity. These findings may facilitate future research endeavors aimed at elucidating the expression and functional roles of the *Cs-g2e3* gene.

## 1. Introduction

In northern China, the Chinese Tongue Sole (*Cynoglossus semilaevis*) is a widely cultivated marine fish, exhibiting a distinct ZW chromosome sex-determining system and apparent sexual dimorphism, where females are typically two to four times larger than males [1,2,3]. However, the sex ratio problem of *Cynoglossus semilaevis* has been an important factor restricting the sustainable development of its aquaculture industry [4]. Females are susceptible to sex reversal by external environmental factors during growth, leading to a decrease in the proportion of females, which affects aquaculture efficiency and market supply [5]. Meanwhile, in the artificial breeding process for Chinese Tongue Sole (*Cynoglossus semilaevis*), the fertilization rate is relatively low, reaching only approximately 25%, necessitating a deeper understanding of its gametogenesis mechanisms [6,7,8]. Therefore, an in-depth exploration of the molecular mechanisms of sex determination and gametogenesis in *Cynoglossus semilaevis* is of great significance in increasing the proportion of females and optimizing the culture structure.

Ubiquitin, a highly conserved 76-amino-acid polypeptide, serves as a key post-translational modifier, attaching to the lysine side chain of substrates through an isopeptide bond at its C-terminal end [9,10]. The process of ubiquitination is a pervasive regulatory mechanism and profoundly affects the functionality and stability of proteins involved in various cellular activities, including protein degradation pathways, signal transduction cascades, and DNA repair mechanisms [11,12,13]. This complex machinery unfolds through an enzymatic cascade involving three distinct enzymes: the ubiquitin-activating enzyme (E1), the ubiquitin-conjugating enzyme (E2), and the ubiquitin ligase (E3) [14]. This cascade begins with the ATP-dependent activation of the E1 enzyme, which forms a thioester bond with the ubiquitin at the cysteine residue in its active site [15]. Then, the ubiquitin is transferred from the E1 to the active site cysteine of the E2 enzyme through a transthioesterification reaction [16]. Then, the E3 ligase orchestrates the crucial transfer of ubiquitin to either the N-terminal primary amine or a lysine side chain of the target protein [17]. By selectively binding to specific substrates, E3 ligases confer substrate specificity, thereby playing a pivotal role in numerous physiological processes, such as gonadal development and morphogenesis [18,19,20].

*G2e3* is a gene encoding a ubiquitin protein ligase, which belongs to a class of enzymes that is categorized into HECT E3, RING E3, and RING-IBR-RING (RBR) E3 enzymes based on their mechanisms for ubiquitin transfer to substrates [21,22]. HECT E3 enzymes, distinguished by a conserved catalytic cysteine residue, function as ubiquitin acceptors from E2 enzymes (E2s), enabling the subsequent transfer of ubiquitin to specific lysine residues on the substrate [23]. Among human ubiquitin ligases, the majority are classified as RING E3 ligases, whereas only 28 types of HECT E3 ligases have been identified, with *g2e3* belonging to the HECT category [21,24]. Disruption of the bifunctional ubiquitin ligase *g2e3*, which is known to inhibit apoptosis during early embryonic stages, results in extensive apoptosis [25]. Wang et al. conducted a phylogenetic analysis and demonstrated that *phf7* in *Drosophila melanogaster* evolved from *g2e3*, a male-specific gene crucial for spermatogenesis in this species [26,27]. Further studies across various species have highlighted the pivotal role of *phf7* in spermatogenesis, with its absence leading to male sterility [28,29,30]. In a transcriptomic comparison between buffalo and cattle, *g2e3* was identified as a novel gene associated with antral follicle apoptosis, suggesting its potential involvement in egg formation [31]. Brooks et al. found that the human testis exhibits the highest expression of *g2e3* mRNA, a tissue known for its extensive physical interactions with multiple proteins [32]. Moreover, Becker et al. reported that the expression levels of *g2e3*, along with *sox6* and *cep41*, were reduced in human males with oligospermia compared to controls [33]. Notably, *g2e3* is also highly expressed in the testis of humans, rats, chickens, zebrafish, and sea urchins, and significant expression has also been observed in the ovary [26].

This study identifies and characterizes the structure and function of *Cs-g2e3* in the *Cynoglossus semilaevis* through comprehensive expression profiling, sequence analysis, in vitro RNA interference experiments, in situ hybridization experiments, and promoter activity assessments. Our findings highlight the crucial role of *Cs-g2e3* in gametogenesis, suggesting its potential as a novel genetic tool that could significantly advance artificial reproduction technologies in aquaculture.

## 2. Materials and Methods

### 2.1. Animal Euthanasia and Ethics Statement

Before conducting the experiments, individual fish were anesthetized with MS-222 to minimize any discomfort. This animal-based research underwent thorough scrutiny and obtained the necessary approval from the Animal Care and Use Committee of the Chinese Academy of Fishery Sciences.

### 2.2. Animals and Samples

Chinese Tongue Soles were cultured at the Weizuo aquaculture base in Tangshan, Hebei, China. These fish were housed in closed indoor concrete ponds equipped with an advanced water circulation system and a real-time environmental monitoring system. The water circulation system purifies the aquaculture water through a series of filters, aeration devices, etc., effectively removes wastes, toxic substances, and excessive nutrients, and thus effectively ensures the quality of aquaculture water. The real-time environmental monitoring system can monitor the temperature, salinity, aquaculture environment temperature, and other indicators of the water in order to regulate the aquaculture environment. During the aquaculture process, the water temperature was maintained between 22 and 26 °C.

DNA was extracted from the fins of each Chinese Tongue Sole using the Marine Tissue Genomic DNA Extraction Kit (TIANGEN Biotech, Beijing, China). Genetic sex determination primers (Cs-sex-F, Cs-sex-R) (Table 1) were then used in PCR amplification to target gender-specific DNA regions. The resulting PCR products were visualized via agarose gel electrophoresis to determine the sex of the fish [34].

Ten different tissues (gonad, liver, spleen, kidney, intestine, brain, skin, muscle, gill, and fin) were collected from four individual 1.5-year-old male fish. Additionally, gonads from four female fish and four male fish at various ages (30 days, 50 days, 100 days, 150 days, 8 months, 1 year, 15 months, and 1.5 years) were harvested. For each age and gender group, at least four fish were sampled to ensure biological replication. All samples were immediately flash-frozen in liquid nitrogen and stored at −80 °C until analysis. Gonads designated for in situ RNA hybridization were fixed overnight in 4% (*w*/*v*) paraformaldehyde (PFA) at 4 °C and then transferred to 75% ethanol for preservation. Each analysis of a specific tissue or age/gender cohort included at least four technical replicates of the experimental procedure.

### 2.3. Cloning and Phylogenetic Analysis of Cs-g2e3

PCR primers (*g2e3*-F, *g2e3*-R; Table 1) targeting the *g2e3* gene (Gene ID: 103382084, mRNA ID: XM_008314747.3) were designed using GenBank information and Primer Premier 5.0. Primer specificity was confirmed using the NCBI Primer-BLAST tool (https://www.ncbi.nlm.nih.gov/tools/primer-blast/index.cgi, accessed on 21 March 2023). The coding sequence (CDS) region of *Cs-g2e3* was cloned and validated by sequencing. Conserved domains of *Cs-g2e3* were predicted using SMART (http://smart.embl-heidelberg.de/smart/set_mode.cgi, accessed on 16 April 2023). Homologous protein sequences were identified and compared using NCBI BLAST (https://blast.ncbi.nlm.nih.gov/Blast.cgi, accessed on 25 April 2023). A phylogenetic tree was constructed using the Poisson amino acid evolution model and the neighbor-joining method, employing MEGA7.0 [35]. The resulting phylogenetic tree was visualized and refined using Evolview (http://www.evolgenius.info/evolview/#/treeview, accessed on 26 April 2023).

### 2.4. Expression Pattern of Cs-g2e3 in Different Tissues and Stages of C. semilaevis

The extraction of total RNA was accomplished from diverse tissues of the *Cynoglossus semilaevis*, as well as from gonads spanning various stages, utilizing the Trizol reagent sourced from Ambion (Austin, TX, USA). RNA concentration was measured by spectrophotometry, and RNA quality was assessed with the Pultton DNA/Protein Analyzer P100 (Plextech, Los Gatos, CA, USA). Reverse transcription was carried out using the gDNA Eraser (Takara, Otsu, Japan) and the PrimeScriptTM RT reagent kit. The resultant cDNA was then amplified on an ABI 7500 rapid real-time PCR system (Applied Biosystems, Foster City, CA, USA) using quantitative primers (*g2e3*-RT-F, *g2e3*-RT-R, Table 1) and a concentration gradient dilution to determine amplification efficiency and to check primer specificity. Subsequently, the cDNA was diluted 10-fold, from which 2 μL of cDNA was mixed with 10 μL of Mix, 0.4 μL of Forward Primer, 0.4 μL of Reverse Primer, and 7.2 μL of ddH2O to form a 20 μL reaction system. After that, qPCR was run according to the corresponding protocol. The following protocol was set for qPCR: 95 °C for 10 s, 95 °C for 5 s, 60 °C for 34 s, 72 °C for 30 s, then 40 cycles are performed. Using *β-actin* as the internal reference gene, which was previously validated for stability and suitability in similar experiments [36].

### 2.5. In Situ RNA Hybridization

To localize *Cs-g2e3* expression within gonadal cells (testicular and ovarian germ cells) of *C. semilaevis*, in situ hybridization (ISH) was performed following a previously established protocol [37,38]. Sense and antisense probes specific to the *Cs-g2e3* gene were synthesized using primers listed in Table 1 and applied to tissue sections following paraffin dewaxing, proteinase digestion for increased tissue permeability, prehybridization, and hybridization procedures. After hybridization, the slides were washed to remove unbound probes and then observed and analyzed for signal generation using a fluorescence microscope.

### 2.6. Cell Culture

In this research endeavor, three distinct cell lines were utilized: the ovarian cell line (CO) and testicular cell line (CT) derived from *C. semilaevis*, as well as human embryonic kidney (HEK) 293T cells. The HEK 293T cells were propagated in Dulbecco’s Modified Eagle Medium (DMEM), enriched with 10% fetal bovine serum sourced from Gibco, Canada, under optimal conditions of 37 °C and 5% CO_2_, adhering to guidelines akin to ATCC CRL-3216™ standards (LGC Standards S.a.r.l., Molsheim, France). Meanwhile, both *C. semilaevis* CO cells [39] and *C. semilaevis* CT cells [40] were maintained in an L-15 medium, fortified with 15% fetal bovine serum (FBS) and a supplement of basic fibroblast growth factor (bFGF) at a concentration of 5 ng/mL, sourced from Invitrogen, Carlsbad, CA, USA. These cells were incubated at 24 °C. Prior to transfection, both cell types were seeded in untreated 24-well or 12-well plates at confluency ranging from 60% to 80% and allowed to proliferate for approximately 24 h.

### 2.7. The Knockdown Effect of Cs-g2e3 siRNA in C. semilaevis Gonad Cells

Based on the *Cs-g2e3* mRNA sequences, the siRNA sites (Table 1) were designed and synthesized by Sangon Co., Ltd. (Shanghai, China). By using the riboFECTTM CP Transfection Kit (Ribobio, Guangzhou, China), the negative control (RNAi-NC), positive control (RNAi-cy3), and the siRNAs for *Cs-g2e3* were transfected into *C. semilaevis* testicular and ovarian germ cells. Prior to dispensing the mixture into each well of a 12-well plate, we prepared a solution by diluting 3 μL of 20 μM siRNA with 60 μL of CP buffer, followed by the incorporation of 5 μL of CP reagent. After 48 h transfection, the silencing efficiency of siRNA was evaluated using the previously mentioned qPCR system and protocols for genes associated with ovarian development, *sox9* (NM_001294243.1) [41], *cyp19a* (NM_001294183.1) [42], and genes associated with testicular development, *tesk1* (NM_001319819.1) [43], *piwil2* (NM_001294236.1) [44]. For detailed information about siRNA synthesis and transfection, please see the Supplementary Materials.

### 2.8. In Vivo RNAi-Mediated Cs-g2e3 Knockdown in the Gonads of C. semilaevis

Five individual sexually mature *C. semilaevis* (all male) were randomly selected from a well-maintained stock and subjected to multiple injections of *Cs-g2e3* siRNA directly into the testes at a concentration of 20 μM, with each fish receiving a total of 10 μg siRNA distributed across three injections at 24-h intervals. Seventy-two hours following the final injection, the testes were collected and processed for analysis to assess the effects of the siRNA-mediated knockdown of *Cs-g2e3* on the expression of male-related genes *tesk1* and *piwil2* using qPCR. 

### 2.9. Construction of Promoter Plasmids, the Interaction between Cs-g2e3 and Transcription Factors, and Co-Transfection and Dual Luciferase Assay in C. semilaevis

To elucidate the function of *Cs-g2e3* in the Chinese Tongue Sole more comprehensively, studies were initiated by investigating the gene’s promoter and associated transcription factors [45]. Promoter primers (*g2e3*-P-F, *g2e3*-P-R, Table 1) were designed utilizing NCBI resources, and a 1498 bp upstream sequence of the *Cs-g2e3* promoter was cloned using KOD One polymerase (TOYOBO, Osaka, Japan) following established PCR and cloning protocols. The PCR products were purified and inserted into the pGL3-Basic plasmid using Xhol and HindIII (Promega, Madison, WI, USA) at 37 °C for 3 h, yielding the pGL3-*Cs-g2e3* promoter construct. Furthermore, potential transcription factor binding sites for C/EBPα, POU1F1a, myogenin, YY1, and JunB within the *Cs-g2e3* promoter were predicted using the Promo tool (http://alggen.lsi.upc.es/cgi-bin/promo_v3/promo/promoinit.cgi?dirDB=TF_8.3, accessed on 23 August 2023). Corresponding plasmids with mutated binding sites for myogenin, YY1, and JunB were constructed using a site-directed mutagenesis kit. These promoter and mutated promoter plasmids were transfected into human embryonic kidney (HEK) 293T cells using Lipofectamine 8000 (Beyotime, Shanghai, China), and the cells were cultured under standard conditions. Luciferase activity was assessed 48 h post-transfection using the Dual-Luciferase Reporter Gene Assay Kit (Beyotime, Shanghai, China), and relative luciferase activities were calculated as Firefly/Renilla ratios. The results, presented as cluster bar graphs using Origin 2017, revealed the influence of specific transcription factors on *Cs-g2e3* promoter activity.

### 2.10. Statistical Analysis

The relative expression levels of *Cs-g2e3* mRNA transcripts were quantified using the formula R = 2^−ΔΔCt^. The expression differences among the experimental groups were analyzed by one-way analysis of variance (ANOVA) and Tukey B using the IBM SPSS Statistics 20. In the siRNA experiments, the data were analyzed with SPSS 25.0 (IBM Corp, Armonk, NY, USA) using a *t*-test. Statistical significance of the data for each gene of interest was assessed by comparison with a negative control (NC), and *p*-value < 0.05 was used as the threshold for determining statistical significance.

## 3. Results

### 3.1. The Structural and Phylogenetic Analyses of g2e3 in C. semilaevis

**Figure 1:**
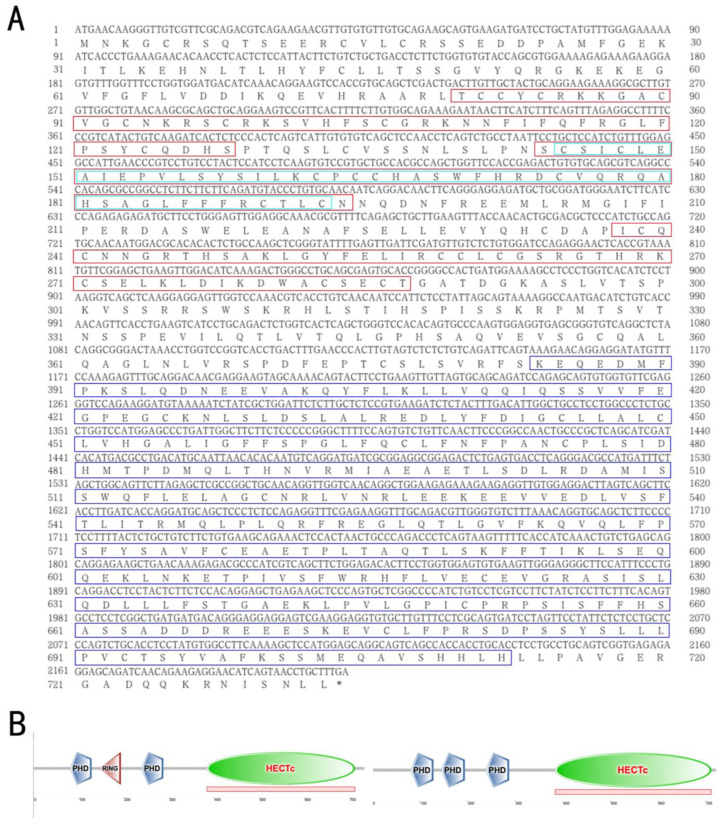

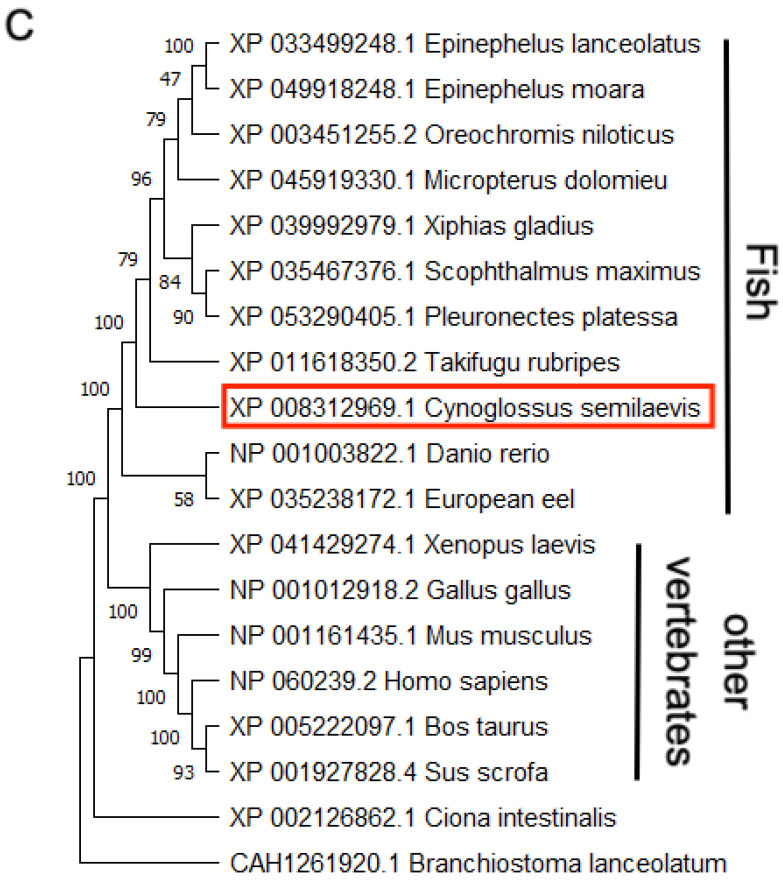
Sequence analysis, conserved structural domain analysis, and phylogenetic tree analysis of *Cs-g2e3* in *C. semilaevis*. Panel (**A**) shows the mRNA sequence and protein amino acid sequence of *Cs-g2e3*. The CDS region of 2202 bp encodes 733 amino acids, with the blue box indicating the conserved HECTc structural domain of the E3 ubiquitin ligase, the red box indicating the conserved PHD structural domain, and the bright green box indicating the conserved RING structural domain. An asterisk (*) represents the stop codon at the end of the ORF. Panel (**B**) displays a map of the conserved structural domains of *Cs-g2e3* predicted by SMART, clearly showing three PHD structures, one HECTc structure, and one RING structure, with the middle PHD and RING structures largely overlapping. Panel (**C**) presents the phylogenetic tree of *g2e3* in fish and other amniotes. *C. semilaevis* is highlighted in red box to indicate its evolutionary position. Numbers at nodes represent NJ bootstrap values. The phylogenetic tree shows that *C. semilaevis* clusters with other fish species, forming a separate group from amniotes such as *Homo sapiens*, *Gallus gallus*, and *Xenopus laevis*.

The genomic sequence of *Cs-g2e3* spans 6479 bp and is mapped to chromosome 1 (NC_024307.1), and includes 17 exons and 16 introns. The total mRNA length of this gene is 3022 bp, encompassing an ORF of 2202 bp that encodes a 733 amino acid protein with a predicted molecular weight of 82.44 kDa and an isoelectric point (PI) of 6.53 (Figure 1A). Predictive analysis of conserved structural domains indicated that the protein contains PHD, Ring, and HECT domains, classifying it as a member of the HECT E3 ubiquitin ligases (Figure 1B). The phylogenetic tree revealed that *C. semilaevis* clusters with other fish species, while amniotes such as *Homo sapiens*, *Gallus gallus*, and *Xenopus laevis* form a separate group (Figure 1C).

### 3.2. Expression Pattern of Cs-g2e3 in C. semilaevis

**Figure 2:**
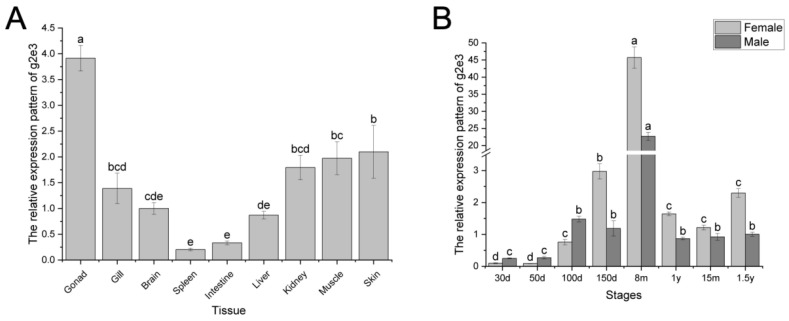
Expression pattern of *Cs-g2e3* in different tissues and stages of *C. semilaevis*. Panel (**A**) represents the expression of *Cs-g2e3* in different tissues, and panel (**B**) represents the expression of *Cs-g2e3* in the gonads of *C. semilaevis* during different stages. The letters above each chart indicate significant differences. The bars represent the standard error.

Quantitative PCR analysis revealed that *Cs-g2e3* expression is detectable in all sampled tissues of *C. semilaevis*, with the highest levels observed in the gonads (Figure 2A). Furthermore, monitoring of the gene’s expression across different stages indicated a gradual increase in expression from 30 days to 8 months, reaching a peak at 8 months of age. Subsequently, a decline in expression was observed between 8 months and 1 year of age, followed by stabilization at 1 year (Figure 2B).

### 3.3. Localization of Cs-g2e3 mRNA in the Gonads of C. semilaevis

**Figure 3:**
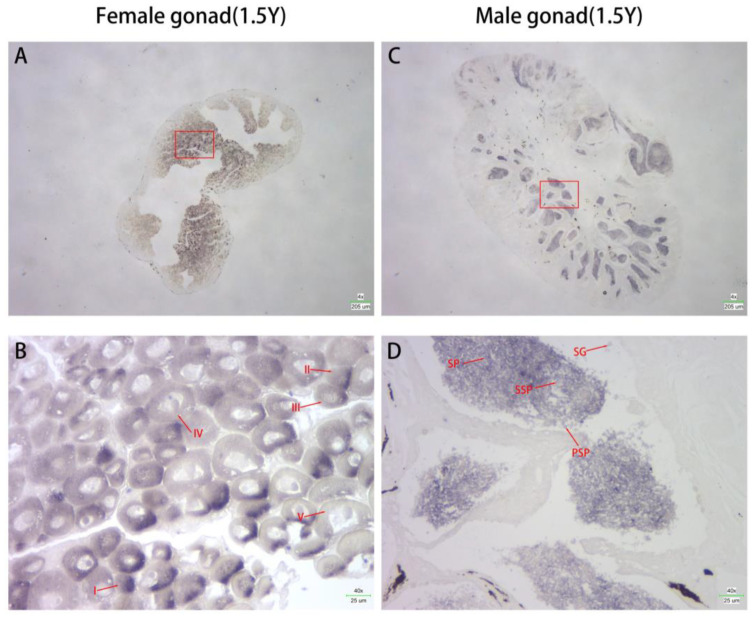
In situ hybridization of *Cs-g2e3* in 1.5-year-old gonads. Panels (**A**,**B**) represent the hybridisation signals of the ovary at different magnifications, and panels (**C**,**D**) represent the hybridisation signals of the testis at different magnifications, and they all show extremely strong hybridisation signals. Oocytes at different developmental stages are marked by I, II, III, IV, and V (panel (**B**)). SG: spermatogonia; PSP: primary spermatocyte; SSP: secondary spermatocyte; SP: spermatozoon (panel (**D**)). Red boxes indicate the same area at different magnifications.

In situ hybridization experiments were conducted to examine the localization of *Cs-g2e3* mRNA in male and female gonads. Results indicated that hybridization signals were predominantly localized in sperm and eggs, with no signals detected in connective or other tissues. Strong hybridization signals were observed in both the sperm and egg of *C. semilaevis* (Figure 3).

### 3.4. The Knockdown Effects on Cs-g2e3 and Other Related Genes by siRNA Transfection in Gonadal Germ Cell Lines

**Figure 4:**
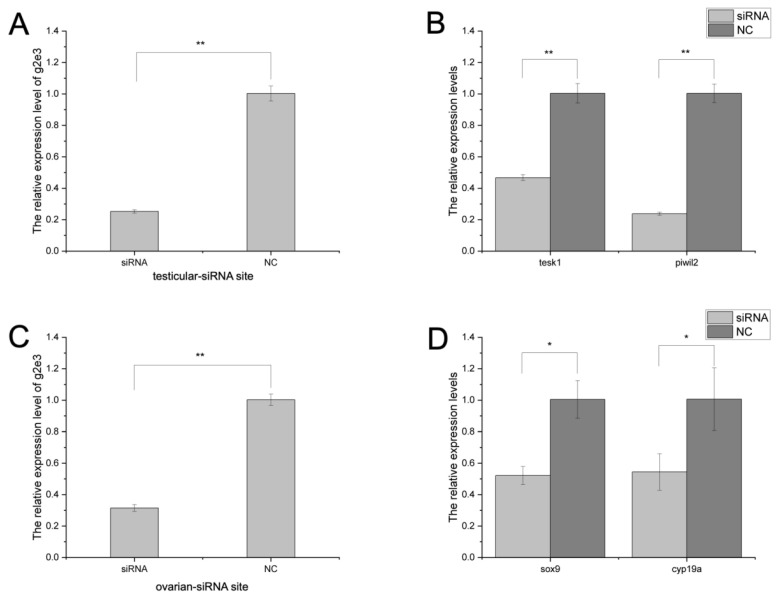
The knockdown effect of *Cs-g2e3* siRNAs in *C. semilaevis* gonadal germ cell lines in mRNA levels. Panel (**A**) shows the gene silencing effect of *Cs-g2e3* siRNA on testicular germ cell lines. Panel (**B**) illustrates the expression patterns of spermatogenesis-related genes, *tesk1,* and *piwil2*, after siRNA transfection. Panel (**C**) shows the gene silencing effect of *Cs-g2e3* siRNA on ovarian germ cell lines. Panel (**D**) illustrates the expression patterns of oogenesis-related genes *sox9* and *cyp19a* after siRNA transfection. The stars indicate a significant difference (**: *p* < 0.01, *: 0.01 ≤ *p* < 0.05). The bars represent the standard error.

In testicular germ cell lines, siRNA knockdown resulted in about a 70% reduction in *Cs-g2e3* transcripts (Figure 4A), with consequent significant downregulation of spermatogenesis-related genes *piwil2* and *tesk1* compared to the negative control (NC) (Figure 4B). In ovarian germ cell lines, siRNA knockdown resulted in about a 70% reduction in *Cs-g2e3* transcripts (Figure 4C), with consequent significant downregulation of oogenesis-related genes *sox9* and *cyp19a* compared to the negative control (NC) (Figure 4D).

### 3.5. In Vivo RNA Interference in C. semilaevis

**Figure 5:**
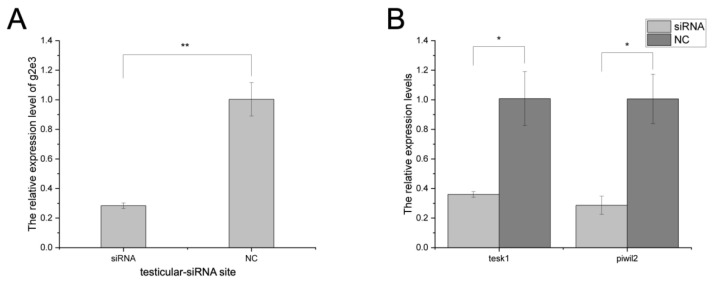
The knockdown effect of *Cs-g2e3* siRNA in testicular tissue of *C. semilaevis* in mRNA levels. Panel (**A**) shows the gene silencing effect of the *Cs-g2e3* siRNA site on the testicular tissue of *C. semilaevis*; panel (**B**) shows the expression of spermatogenesis-related genes *tesk1* and *piwil2* after vivo RNA interference. The stars indicate a significant difference (**: *p* < 0.01, *: 0.01 ≤ *p* < 0.05). The bars represent the standard error.

In vivo RNA interference in the testicular tissue showed that siRNA knockdown was effective, with efficiencies exceeding 70% (Figure 5A). Gene expression of spermatogenesis-related genes *piwil2* and *tesk1* was significantly downregulated compared to the negative control (NC) (Figure 5B), consistent with the results of RNA interference in testicular germ cell lines.

### 3.6. Detection and Analysis of the Activity of the Cs-g2e3 Promoter and the Regulation of Transcription Factors

**Figure 6:**
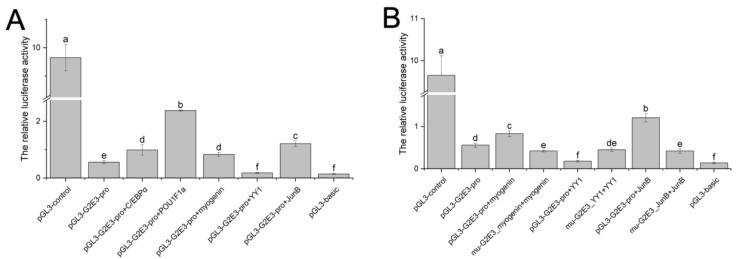
Analysis of the activity of the *Cs-g2e3* promoter and the regulatory roles of transcription factors. Panel (**A**) shows the activity of the *Cs-g2e3* promoter and the effects of co-transfection with transcription factors C/EBPα, POU1F1a, myogenin, YY1, and JunB on its activity. Panel (**B**) shows the effects of mutations in the transcription factor binding sites for myogenin, YY1, and JunB on *Cs-g2e3* promoter activity. In the one-way ANOVA, different letters are used to indicate statistically significant differences among the groups. The bars represent the standard error.

Dual luciferase assays revealed robust activity of the *Cs-g2e3* promoter (Table 2). When co-transfected with transcription factors C/EBPα, POU1F1a, myogenin, and JunB, the promoter activity was significantly enhanced, whereas co-transfection with YY1 markedly reduced it to levels comparable to those observed with the pGL3-Basic vector (Figure 6A). Mutations in the transcription factor binding sites for myogenin and JunB resulted in a significant decrease in *Cs-g2e3* promoter activity upon co-transfection, highlighting the activating functions of these factors. Conversely, mutation of the YY1 binding site led to a significant increase in promoter activity, indicating a repressive role for YY1 in regulating *Cs-g2e3* promoter activity (Figure 6B). These findings suggest that YY1 may serve as a pivotal regulator of *Cs-g2e3* transcription.

## 4. Discussion

Ubiquitination, a ubiquitous post-translational modification, plays a pivotal role in regulating a myriad of cellular processes, notably including the development of mammalian ovarian and testicular germ cells [11,46]. In both amniotes and fish, the levels of ubiquitination undergo a marked increase during gonadal differentiation and gametogenesis [47,48]. Analogously, the ubiquitin ligase gene, designated as *g2e3*, has been observed to exhibit high levels of expression within the gonads across diverse species, such as humans, rats, chickens, zebrafish, and sea urchins [26]. In the present study, the *g2e3* gene was likewise found to exhibit high expression levels in the gonads of Chinese Tongue Sole.

In our study, we successfully cloned *Cs-g2e3*, determined its sequence, and analyzed its structural features, confirming that *Cs-g2e3* belongs to the canonical HECT E3 ligase family, in line with observations made in other species [21,49]. RT-PCR analysis revealed that *Cs-g2e3* was predominantly expressed in gonadal tissues, lending support to our hypothesis that *Cs-g2e3* is intricately involved in gametogenesis. Further analysis revealed that *Cs-g2e3* expression peaked at 8 months of age, subsequently declining from 8 months of age to 1 year of age and stabilizing thereafter. A prior study indicated that sexual differentiation in *C. semilaevis* occurs around 60 days, which may underlie the initial low expression of *Cs-g2e3* [41]. Subsequently, male and female gonads differentiate and develop, producing gametes. By 8 months, the gonads are primarily mature, coinciding with the peak *Cs-g2e3* expression. After maturity, the production of gametes is in a dynamic equilibrium, which is consistent with the results of the stabilization of the expression of *Cs-g2e3* [50,51]. We also found that *Cs-g2e3* expression was higher in male gonads than in female gonads between 30 and 100 days of age and lower in male gonads than in female gonads thereafter, with the most significant difference at 8 months of age, which may be due to sexual dimorphism in *C. semilaevis*. In situ hybridization experiments conducted on both male and female *C. semilaevis* specimens yielded strong *Cs-g2e3* hybridization signals within the testis and ovary, primarily localized to spermatozoa and eggs, respectively. These findings were reinforced by siRNA knockdown experiments performed in gonadal germ cell lines derived from *C. semilaevis*. These experiments targeted genes previously implicated in gametogenesis, including *tesk1*, *piwil2*, *sox9*, and *cyp19a* [41,42,43,44]. Notably, the knockdown of *Cs-g2e3* significantly altered the expression patterns of these genes, thereby confirming *Cs-g2e3’*s functional role in gametogenesis. This conclusion was further validated in vivo through the injection of siRNA directly into the testis of *C. semilaevis*, yielding results that were consistent with those observed in the germ-cell line experiments.

We also evaluated the activity of the *Cs-g2e3* gene promoter and found it to be highly active. By predicting and mutating its binding sites, we observed that the *Cs-g2e3* promoter affects these sites to varying degrees, with a particularly strong association with the YY1 transcription factor. This suggests that YY1 may be a key transcription factor that initiates *g2e3* gene expression. As a transcription factor, YY1 plays a crucial role in gonadal development and spermatogenesis in many mammals. In studies on chicken testis, YY1 was shown to regulate gonadal development and mediate spermatogenesis and differentiation [52,53,54]. Furthermore, localization of YY1 in the adult testis revealed that it is predominantly present in the nuclei of spermatogonia and spermatozoa, with a particularly strong signal detected in spermatogonia [55]. Similarly, studies in mice have shown the involvement of YY1 in spermatogenesis [56,57]. In addition, YY1 is involved in gonadal development and gametogenesis in fish, such as in yellow catfish, where YY1 can act as a marker of egg quality and play an important role in oogenesis [58], in *Pimephales promelas*, YY1 is also involved in ovarian development and regulates the synthesis of sex hormones [59]. More importantly, a previously reported study on YY1 in *C. semilaevis* showed that YY1 is involved in gonadal development in *C. semilaevis*, which may include gametogenesis [60]. From the experimental results, it was observed that the binding of YY1 to the *Cs-g2e3* promoter led to a significant decrease in its activity. Conversely, mutation of the YY1 binding site restored the promoter activity to its original level, further confirming that YY1 exerts an inhibitory effect on the *Cs-g2e3* promoter. Previous studies have indeed established that YY1, a crucial transcription factor implicated in spermatogenesis, is localized within spermatogonial stem cells and influences this process by participating in meiosis [55,57]. However, the regulatory mechanisms involved in gametogenesis in *C. semilaevis* deserve further investigation. Thus, our studies on the *Cs-g2e3* promoter and transcription factors further emphasize the possible involvement of *Cs-g2e3* in the physiological process of spermatogenesis. This finding serves as a valuable reference for subsequent in-depth studies on transcription factors related to gametogenesis. Meanwhile, given the current low fertilization rate of *C. semilaevis*, we are considering the possibility of injecting relevant *Cs-g2e3* carrier proteins in future production practices of *C. semilaevis* culture. This would potentially allow the spermatophore to continue spermatogenesis and produce viable sperm for artificial insemination, thereby improving the fertilization rate and promoting the development of the *C. semilaevis* culture industry. Furthermore, this approach offers valuable ideas for future breeding work and production of *C. semilaevis*.

## 5. Conclusions

In this study, we successfully cloned and characterized the cDNA of the ubiquitin ligase gene *g2e3* in *C. semilaevis* (*Cs-g2e3*). *Cs-g2e3* was predominantly expressed in the gonads compared to other tissues. The gene’s expression increased progressively and reached its peak at 8 months of age, then declined and stabilized, maintaining levels significantly higher than those observed in the juvenile period. In situ hybridization demonstrated that *Cs-g2e3* was mainly localized in the germ cells. In vitro RNAi studies showed that knockdown of *Cs-g2e3* in ovarian and testicular germ cell lines significantly downregulates the expression of key genes involved in oogenesis (e.g., *sox9* and *cyp19a*) and spermatogenesis (e.g., *tesk1* and *piwil2*), respectively. Additionally, in vivo experiments confirmed that the injection of siRNA into the testis of *C. semilaevis* similarly affected the expression of *tesk1* and *piwil2*. Experiments exploring the regulation of the *Cs-g2e3* promoter and its interaction with transcription factors identified a significant relationship between the transcription factor YY1, associated with spermatogenesis, and the *Cs-g2e3* promoter. These findings underscore the involvement of *Cs-g2e3* in gametogenesis and highlight its critical role in the reproductive biology of *C. semilaevis*.

## Figures and Tables

**Table 1:** Primers used in experiments.

Primer Name	Sequence (5′ to 3′)	Product Length (bp)	Tm Values (°C)	Purposes
*g2e3*-F	GCCACGTAACCCCATAT	2869	52	sequence validation
*g2e3*-R	AACGCAAGCAGTAGAACAG			
*β-actin*-RT-F	TTCCAGCCTTCCTTCCTT	124	53	qRT-PCR
*β-actin*-RT-R	TACCTCCAGACAGCACAG			
*g2e3*-RT-F	GTGTTCGAGGGTCCAGAAGG	126	58	qRT-PCR
*g2e3*-RT-R	GAAGCCAATCAGGGCTCCAT			
Y-*g2e3*-F	ATTTAGGTGACACTATAGAA CTGCAGGAAGTCCGTTCACT	556	65	ISH
Y-*g2e3*-R	TAATACGACTCACTATAGGG GGCCCAGTCTTTGATGTCCA			
*sox9*-RT-F	AAGAACCACACAGATCAAGACAGA	150	57	qRT-PCR
*sox9*-RT-R	TAGTCATACTGTGCTCTGGTGATG			
*cyp19a*-RT-F	GGTGAGGATGTGACCCAGTGT	230	56	qRT-PCR
*cyp19a*-RT-R	ACGGGCTGAAATCGCAAG			
*tesk1*-RT-F	GCAGAAACTCTCTCACCCCAACA	290	59	qRT-PCR
*tesk1*-RT-R	CCAGACCAAAGTCCGTCACCA			
*piwil2*-RT-F	CGTCACCTTCGCTCCAAAT	171	56	qRT-PCR
*piwil2*-RT-R	TCTTCGTCGTCCGTTCGC			
*g2e3*-P-F	AGATCTGCGATCTAAGTAAGCT GCGTCCTCCAGTTTGGCTA	1540	68	Promoter cloning
*g2e3*-P-R	CAACAGTACCGGAATGCCAAGCT CGCTTTTCTTCCGTTCCG			
Cs-sex-F	CCTAAATGATGGATGTAGATTCTGTC	169/134	56	sex determination
Cs-sex-R	GATCCAGAGAAAATAAACCCAGG			
*g2e3*-835-F	GCAACAAUCAGGACAACUUTT			siRNA
*g2e3*-835-R	AAGUUGUCCUGAUUGUUGCTT			
*g2e3*-1562-F	CCGUGAAGAUCUCUACUUUTT			siRNA
*g2e3*-1562-R	AAAGUAGAGAUCUUCACGGTT			
*g2e3*-1928-F	GCAGACGUUGGGUGUCUUUTT			siRNA
*g2e3*-1928-R	AAAGACACCCAACGUCUGCTT			

**Table 2:** The luciferase values in human embryonic kidney (HEK) 293T cells.

Groups	Firefly Luciferase	Renilla Luciferase	The Relative Luciferase Value (±SD)
pGL3-*g2e3*-pro	640,038	1,102,383	0.56 ± 0.05
	571,785	954,903	
	511,223	1,016,504	
pGL3-*g2e3*-p + C/EBPα	640,123	586,912	0.99 ± 0.18
	706,702	644,511	
	391,717	499,763	
pGL3-*g2e3*-p + POU1F1a	941,729	395,459	2.38 ± 0.02
	786,581	327,948	
	984,677	417,072	
pGL3-*g2e3*-p + myogenin	812,538	1,023,484	0.83 ± 0.07
	868,884	950,887	
	883,088	1,132,129	
pGL3-*g2e3*-p + YY1	365,876	1,897,689	0.18 ± 0.02
	305,774	1,580,501	
	321,856	1,940,210	
pGL3-*g2e3*-p + JunB	143,5884	1,269,235	1.21 ± 0.10
	134,7471	1,020,311	
	1,462,650	1,236,206	
mu-*g2e3*_myogenin + myogenin	841,408	1,980,682	0.42 ± 0.02
	851,162	1,927,374	
	742,405	1,831,696	
mu-*g2e3*_YY1 + YY1	1,565,732	3,620,141	0.45 ± 0.04
	1,682,182	3,401,520	
	1,014,972	2,447,655	
mu-*g2e3*_JunB + JunB	339,348	854,085	0.42 ± 0.05
	293,453	623,706	
	356,566	937,653	
pGL3-control	7,175,562	766,433	9.65 ± 0.47
	9,262,670	909,082	
	12,162,782	1,294,003	
pGL3-Basic	105,484	765,987	0.14 ± 0.02
	107,500	648,147	
	139,058	1,061,613	

## Data Availability

The data presented in this study are available in this article.

## Supplementary Materials

The following supporting information can be downloaded at: https://www.mdpi.com/article/10.3390/ani14172579/s1, *g2e3* siRNA synthesis and transfection.
