# Supplementary material for: Identification and Functional Analysis of E3 Ubiquitin Ligase g2e3 in Chinese Tongue Sole, Cynoglossus semilaevis
Source: Animals (Basel). 2024 Sep 5;14(17):2579. doi: 10.3390/ani14172579 (PMC11394658; doi:10.3390/ani14172579)
Supplement: Supplementary file 1 [file animals-14-02579-s001.zip › animals-3145900-supplementary.pdf]

**Supplementary Information**

# Identification and Functional Analysis of E3 Ubiquitin Ligase *g2e3* in Chinese Tongue Sole, *Cynoglossus Semilaevis*

**Zhongkai Cui [1,2,†], Jun Luo [1,2,3,†,‡], Fangzhou Cheng [1,2,3], Wenteng Xu [1,2], Jialin Wang [1,2,3], Mengjiao Lin [1,2,3], Yuqi Sun [1,2] and Songlin Chen [1,2,3,*]**

[1] State Key Laboratory of Mariculture Biobreeding and Sustainable Goods, Yellow Sea Fisheries Research Institute, Chinese Academy of Fishery Sciences, Qingdao 266071, China

[2] Laboratory for Marine Fisheries Science and Food Production Processes, Qingdao Marine Science and Technology Center, Qingdao 266237, China

[3] College of Fisheries and Life Science, Shanghai Ocean University, Shanghai 201306, China

* Correspondence: chensl@ysfri.ac.cn; Tel.: +86-(0)532-85831605

† These authors contributed equally to this work.

‡ Current address: Pingxiang Aquatic Research Institute, Pingxiang Agricultural Science Research Center, Pingxiang 337099, China.

## Supplementary Information

### I. siRNA synthesis

**Method selection:** In our study, siRNA was synthesized by chemical synthesis method due to its high specificity, stability and reproducibility. This method ensures the effectiveness and targeting of siRNA by precisely controlling the synthesis of RNA sequences.

**Chemical reagents and service providers:** We selected Sangon Co., Ltd. (Shanghai, China) for the custom synthesis of siRNA. The company ensured the accuracy and purity of the siRNA sequences. All siRNA molecules were purified by HPLC and their sequence correctness was verified by mass spectrometry.

**Sequence information:**

| Primer name | sequence (5′ to 3′) |
|---|---|
| *g2e3*-835-F | GCAACAAUCAGGACAACUUTT |
| *g2e3*-835-R | AAGUUGUCCUGAUUGUUGCTT |
| *g2e3*-1562-F | CCGUGAAGAUCUCUACUUUTT |
| *g2e3*-1562-R | AAAGUAGAGAUCUUCACGGTT |
| *g2e3*-1928-F | GCAGACGUUGGGUGUCUUUTT |
| *g2e3*-1928-R | AAAGACACCCAACGUCUGCTT |

## II. siRNA transfection

**Transfection reagent:** We chose riboFECTTM CP Transfection Kit (Ribobio, Guangzhou, China) as the transfection reagent. This reagent is known for its high efficiency, low toxicity and widely applicable cell types, and has been widely used in siRNA transfection experiments for a variety of cells.

**Cell type:** This study used the ovarian cell line (CO) and testicular cell line (CT) of *Cynoglossus semilaevis.*

**Transfection concentration:** Prior to dispensing the mixture into each well of a 12-well plate, we prepared a solution by diluting 3 μL of 20 μM siRNA with 60 μL of CP buffer, followed by the incorporation of 5 μL of CP reagent.

**Transfection time:** siRNA was mixed with transfection reagents and added into the cell culture medium, and continued to incubate for 48 hours to achieve the best transfection effect.

**Evaluation of transfection efficiency:** We evaluated the transfection efficiency of siRNA and the knockdown effect on *Cs-g2e3* genes by qPCR method. The results showed that the selected transfection conditions were able to effectively introduce siRNA into the target cells and significantly down-regulate the expression level of the *Cs-g2e3* genes, and the knockdown efficiencies all reached more than 70%.
